# The Inhibitory Effect of Connective Tissue Growth Factor Antibody on Postoperative Fibrosis in a Rabbit Model of Trabeculectomy

**DOI:** 10.18502/jovr.v17i4.12300

**Published:** 2022-11-29

**Authors:** Kiana Hassanpour, Mozhgan Rezaei Kanavi, Narsis Daftarian, Azadeh Samaeili, Fatemeh Suri, Mohammad Pakravan, Azadeh Doozandeh, Sasha Afsar Aski, Maryam Fakhri, Afrooz Moghaddasi, Hamid Ahmadieh, Hamed Esfandiari

**Affiliations:** ^1^Ophthalmic Research Center, Research Institute for Ophthalmology and Vision Science, Shahid Beheshti University of Medical Sciences, Tehran, Iran; ^2^Department of Ophthalmology, Imam Hossein Hospital, Shahid Beheshti University of Medical Sciences, Tehran, Iran; ^3^Ocular Tissue Engineering Research Center, Research Institute for Ophthalmology and Vision Science, Shahid Beheshti University of Medical Sciences, Tehran, Iran; ^4^Ophthalmic Epidemiology Research Center, Research Institute for Ophthalmology and Vision Science, Shahid Beheshti University of Medical Sciences, Tehran, Iran; ^5^Department of ophthalmology, Olmsted Medical Center, Rochester, MN, USA; ^7^Kiana Hassanpour: 0000-0002-1788-7352; ^8^Hamed Esfandiari: 0000-0001-7301-7047

**Keywords:** Anti-connective Tissue Growth Factor, CTGF, Mitomycin-C, Trabeculectomy

## Abstract

**Purpose:**

To compare the efficacy of subconjunctival injection of an anti-connective tissue growth factor antibody (anti-CTGF) versus mitomycin-C (MMC) and placebo in reducing scar formation in a rabbit model of trabeculectomy.

**Methods:**

A total of 14 rabbits were included. Nine rabbits underwent trabeculectomy with subconjunctival injections of either anti-CTGF antibody, MMC, or balanced salt solution (BSS), each administered in three eyes, before peritomy. The anti-CTGF group received a repeated dose of the antibody five days after surgery. All nine rabbits were euthanized on day 14; the globes were stained with hematoxylin & eosin, Masson's Trichrome, and immunohistochemistry for detecting alpha-smooth muscle (α-SMA) actin. RNA extraction was performed on five eyes of the remaining rabbits which included one eye without any surgery, one eye 5 hr after trabeculectomy without any injection, one eye five days after trabeculectomy without any injection, and two eyes five days after trabeculectomy with administration of MMC and BSS, respectively.

**Results:**

The mean bleb area in the anti-CTGF, MMC, and control groups was 3.8 ± 1.45, 5.9 ± 1.4, and 3.5 ± 1.9 mm^2^, respectively. Collagenous tissue was found to occupy the bleb area by 13.7%, 13.5%, and 18.5%, respectively. This ratio was significantly higher in the BSS group (*P* = 0.04). The expression of CTGF mRNA after 5 hr and five days in eyes undergoing trabeculectomy were significantly more pronounced as compared to the unoperated eye. The mean H-SCORE of α-SMA-immune reactive cells calculated as the grade of staining multiplied by the percentage of immune stained cells was 14.6, 10.22, and 140.58 in the anti-CTGF, MMC, and control groups, respectively. While the control eyes had a significantly higher score (*P*s < 0.001), the anti-CTGF and MMC groups were comparable (*P* = 0.87).

**Conclusion:**

Based on the results of this animal study, the anti-CTGF antibody injection resulted in a significant reduction in collagenous tissue and myofibroblast cells after trabeculectomy.

##  INTRODUCTION

Trabeculectomy is the most common surgical procedure for eyes with severe glaucoma in which intraocular pressure (IOP) at low teens is required.^[1]^ The main reason for failure of trabeculectomy is obstruction of aqueous drainage through the site of surgery. Even with newer surgical techniques, scarring and fibrosis compromise the long-term results of trabeculectomy. Antifibrotic agents have revolutionized the outcomes of trabeculectomy; however, their nonspecific mechanisms of action result in potentially blinding complications which call for new interventions with an improved safety profile.^[2,3,4]^

Wound healing is mediated by several cell types and is coordinated by a complex array of cytokines, chemokines, and growth factors. Transforming growth factor-beta (TGF-β) plays an essential role in the post-trabeculectomy wound healing process; however, attempts to use TGF-β inhibitors to reduce fibrosis following trabeculectomy have not been successful.^[5]^ Connective tissue growth factor (CTGF) is a downstream mediator for the fibrotic activity of TGF-β and is expressed in fibroblasts, smooth muscle fibers, or endothelial cells. CTGF belongs to the family of extracellular matrix (ECM)-associated proteins, that is, CYR61 (cysteine-rich angiogenic protein 61 or CCN1), CTGF (connective tissue growth factor or CCN2), and NOV (nephroblastoma overexpressed or CCN3), collectively referred to as the CCN family. It has several biological roles in all fibrosis processes.^[6,7,8,9,10,11]^ Yuan et al showed that CTGF is overexpressed in the filtering bleb suggesting evidence of its role in the post-trabeculectomy wound healing process.^[12]^ Subsequently, Wang et al^[13]^ showed a larger bleb area and lower IOP after a subconjunctival injection of an anti-CTGF antibody after trabeculectomy in an experimental study. CTGF has also been shown to be present at higher concentrations in the aqueous humor of eyes with different types of glaucoma.^[13,14,15,16,17]^

Unlike antimetabolites, monoclonal antibodies have the advantage of focusing on specific targets in the tissue which limits their toxicity and makes them suitable for a multi-therapeutic approach. The current experimental study was conducted to compare the effect of a subconjunctival injection of anti-CTGF antibody on collagen tissue formation and myofibroblasts of the bleb in comparison with MMC-treated and control groups after trabeculectomy in a rabbit model.

##  METHODS

All experimental procedures in this study were approved by the Ethics Committee of the Ophthalmic Research Center at Shahid Beheshti University of Medical Sciences, Tehran, Iran (Code number: IR.SBMU.ORC.REC.1397.17).

### Animal Preparation and Grouping

Fourteen healthy female New Zealand albino rabbits were taken from Razi Institute for Vaccine and Serum Research, Karaj, Iran. The rabbits were between 10 and 12 months of age, weighed 2–3 kg, were kept under standard conditions (temperature 20 ± 1ºC, 12-hr light–dark cycle), and received care as mentioned in the ARVO Statement for the Use of Animals in Ophthalmic and Vision Research. All animals were checked in terms of corneal, lenticular, and vitreous clarity and were then randomized into three groups of three animals (three eyes) each; namely anti-CTGF, MMC, and balanced salt solution (BSS) groups. Five eyes of five rabbits were used for RNA extraction.

### Surgical Technique

To provide anesthesia, an intramuscular injection of 10% ketamine HCL (Alfamine; 50 mg/kg; Alfasan, Woerden, Holland) and Xylazine (Rompun; 5 mg/kg; Bayer, Leverkusen, Germany) was used. After randomization of the rabbits and using a topical 0.5% tetracaine eye drop (Anestocaine, Sina Darou Laboratories, Tehran, Iran) in the rabbits' right eyes, an anti-CTGF antibody (0.1 ml of 250 µg/mL), MMC-C (0.1 mL of 0.2 mg/ml for 3 min followed by irrigation), and BSS (0.1 mL) were injected into the subconjunctival space at the presumed site of the bleb. In this study, the anti-human CTGF neutralizing antibody (Peprotech, NJ, USA) was diluted to 250 µg/mL in a sterile fashion as previously described.^[18]^ The surgeon was masked to the injection type before and after the procedure.

Immediately after the injection, a standard fornix-based trabeculectomy was performed by an experienced surgeon (AS). A 6-mm fornix-based conjunctival incision was made and the underlying tenon's capsule was dissected toward the fornix. Then a 3 × 2.5 × 2.5 mm trapezoidal half-thickness scleral flap was created using a crescent knife and lamellar dissection of the scleral flap 1-mm into clear cornea was performed. After making a paracentesis, a keratome was used to enter the anterior chamber underneath the scleral flap, and an anterior tissue block containing the inner sclera, trabecular meshwork (TM), and peripheral cornea measuring approximately 1.5 × 1 mm was removed with a Kelly punch. Peripheral iridectomy (PI) was performed using Vannas scissors. The scleral flap and overlying conjunctiva were closed with a 2 × 10-0 nylon and 8/0 Vicryl sutures, respectively. Postoperatively, each rabbit received topical chloramphenicol 0.5% eye drops (Sina Darou Lab, Tehran, Iran) for 14 days. Topical steroids were not used in the study to evaluate the primary effect of the anti-CTGF in comparison with MMC and BSS. Since it has been shown that the highest concentration of CTGF occurs at five days postoperatively,^[8]^ the second subconjunctival injection of anti-CTGF antibody (0.1 ml of 250 µg/mL) was done on the fifth-day follow-up. At this time, the anti-CTGF was injected without advancing the needle into the bleb or under the scleral flap. Postoperatively, eyes were examined for corneal clarity, depth of the anterior chamber, roundness of pupil, and formation of trabeculectomy blebs with a slit-lamp biomicroscope.

### Histopathology and Immunohistochemistry

After euthanizing the animals with intracardiac injections of 1 mL pentobarbital sodium on day 14 after surgery, the whole eyes were removed and fixed in 10% buffered formalin. The middle calotte including the whole trabeculectomy site was then subjected to tissue processing and embedding into paraffin blocks. Consecutive thin sections at three different tissue levels (250 µm apart) from the operative wound site (indicated by the PI site) were prepared and stained with hematoxylin and eosin (H&E) and Masson's trichrome staining. An ocular pathologist (MRK), who was masked to the study groups, examined the stained slides under light microscopy (BX41, Olympus, Japan) in an effort to analyze the trabeculectomy blebs and blue-stained collagenous tissue. Corresponding photographs were also captured with a digital camera (DP12 Microscope Camera, Olympus, Japan). Image J software (Image J 1.48, National Institute of Mental Health; http://rsb.info.nih.gov/ij/) was then used to quantify the corresponding areas. Three slides were analyzed per bleb. The ratio of the blue-stained collagenous area to the area of the trabeculectomy bleb was also calculated.

The presence of myofibroblastic cells in the trabeculectomy blebs was identified using immunohistochemistry to detect α-smooth muscle actin (α-SMA). Tissue sections were sequentially subjected to antigen retrieval, introducing a blocking agent, overnight incubation with a mouse anti-α-SMA monoclonal antibody (1:200, Abcam, Cambridge, UK) at 4ºC, and then 45 min-incubation with a fluorescein Isothiocyanate (FITC)-conjugated goat anti-mouse IgG (1:200; Abcam, Cambridge, UK) at room temperature in the dark. After counterstaining the cell nuclei with 4',6-diamidino-2-phenylindole (DAPI) (1 mg/ml; Santa Cruz Biotechnology Inc., Dallas, USA) for 5 min, the stained sections were examined by fluorescence microscopy (Olympus IX71; Tokyo, Japan). Photomicrographs of the trabeculectomy blebs were captured with a digital camera (Olympus U-TV0.63XC; Tokyo, Japan). The α-SMA-immune reactive cells in the blebs were scored by calculating the “H-SCORE” (H-SCORE = the grade of immunohistochemical staining [I] × the percentage of immune stained cells [PC]). The immunohistochemical staining was graded semi-quantitatively as follows: 0 = no staining; 1 = weak staining; 2 = moderate staining; and 3 = strong staining.^[19]^ The ratio of green fluorescent cells with DAPI-stained nuclei to all DAPI-stained nuclei was considered as PC. The grader was masked to the study group.

### mRNA Extraction and Quantitative Real-time Polymerase Chain Reaction (PCR)

To assess the expression of CTGF mRNA in the bleb area and compare its expression at different time intervals after trabeculectomy, total RNA was extracted from five different rabbit eye groups which included one intact eye without any surgery, one eye 5 hr after trabeculectomy, one eye five days after trabeculectomy with no injection, one eye five days after trabeculectomy with the administration of a BSS injection, and finally one eye five days after trabeculectomy with MMC injection. The globe sections including the trabeculectomy areas in the operated eyes were homogenized and the AccuZol total RNA extraction kit (K-3090, Bioneer, Korea) was used to extract the total RNA. The concentration/purity and the integrity of the isolated RNA were determined using a NanoDrop instrument (Thermo Scientific, Waltham, MA, USA) and agarose gel electrophoresis, respectively. cDNA was then generated by reverse transcription of the total RNA using a Revert Aid First Strand cDNA Synthesis Kit (#K1621, Thermo Scientific, Waltham, MA, USA). Subsequently, real-time PCR was performed utilizing a Corbett 65H0 machine (Corbett Research, Sidney, Australia) using the SinaSYBR Blue HS-qPCR Mix (#MM2171, Sinaclon, Tehran, Iran). B2M (beta-2 microglobulin) gene expression was quantified as the reference gene. Real-time PCR primers sequences used in this study are listed in Table 1.

### Statistical analysis

To describe the data, we used mean and standard deviation (SD). To evaluate the differences between the study groups, Kruskal–Wallis and Mann–Whitney U tests were used. Statistical analyses were performed using the SPSS software (IBM Corp. released in 2013, IBM SPSS Statistics for Windows, Version 24.0, Armonk, NY: IBM Corp). For statistical analysis of real-time-PCR data, one-way analysis of variance (ANOVA) with Kruskal–Wallis post-comparison test was performed using the Prism software package (GraphPad; https://www.graphpad.com/; significance at < 0.05, < 0.01, and < 0.001 were indicated in figures by *, **, and ***, respectively.) Results were considered statistically significant at a *P*-value of < 0.05.

**Table 1:** Sequences of the designed primers.


**Gene**	**Forward primer**	**Reverse primer**
***CTGF***	CTGGCCGCCTACCGACTG	TCTCTTCCAGGTCAGCTTCG
***B2M***	CAGCGTGCTCCGAATGTTC	GTAATCTCGATCCCATTTCAC
	
	
CTGF, connective tissue growth factor; B2m, beta-2-microglobulin gene		

**Figure 1:**
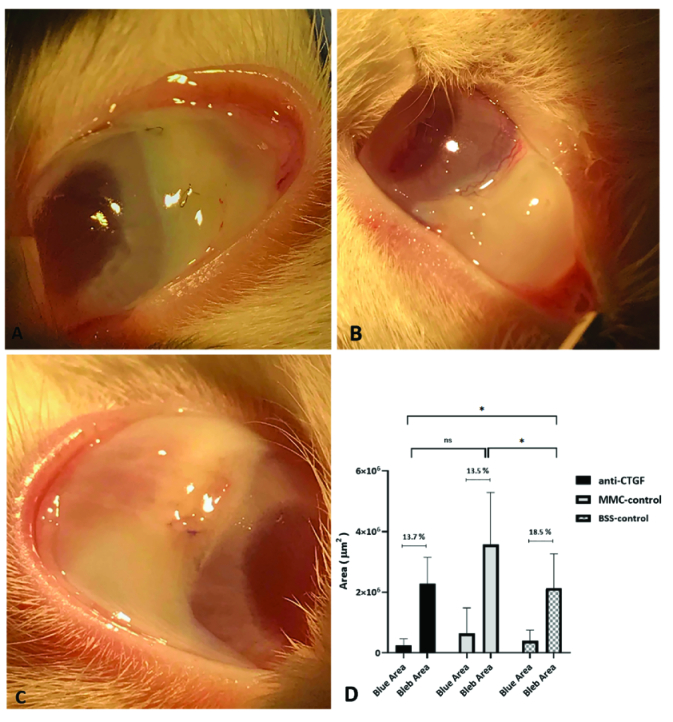
Representative images of trabeculectomy blebs in the study groups. Note the presence of well-formed blebs in the anti-CTGF (A), MMC (B), and control (C) eyes on day 14 postoperatively. (D) Bleb area compared with the blue area in anti-CTGF, MMC, and BSS groups, respectively.

**Figure 2:**
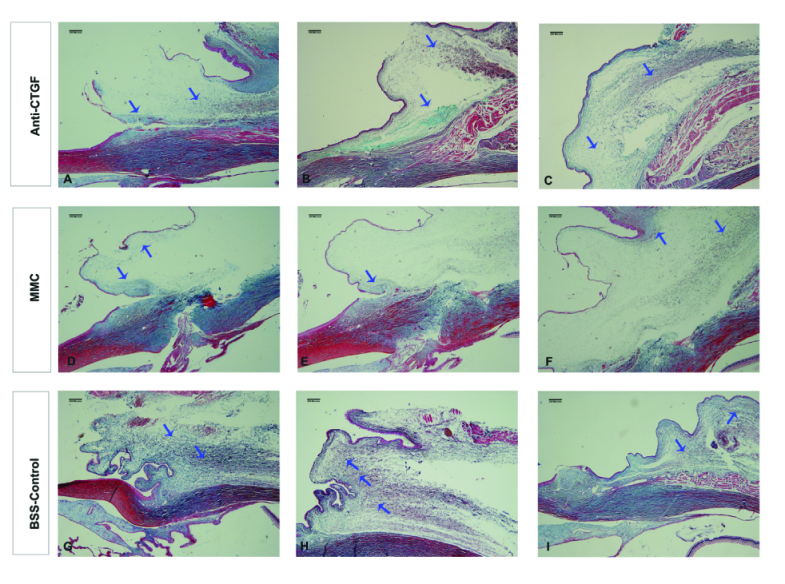
Representative photomicrographs of trabeculectomy blebs in the study groups. Note the lower content of blue-stained collagenous tissue in the trabeculectomy blebs of the anti-CTGF (A, B, C), MMC (D, E, F), and control (G, H, I) eyes. Blue arrows point to the more prominent, blue-stained collagenous tissue in the bleb areas. Other diffused, blue-stained areas were also entered in the Image J analysis (Masson's trichrome staining).

**Figure 3:**
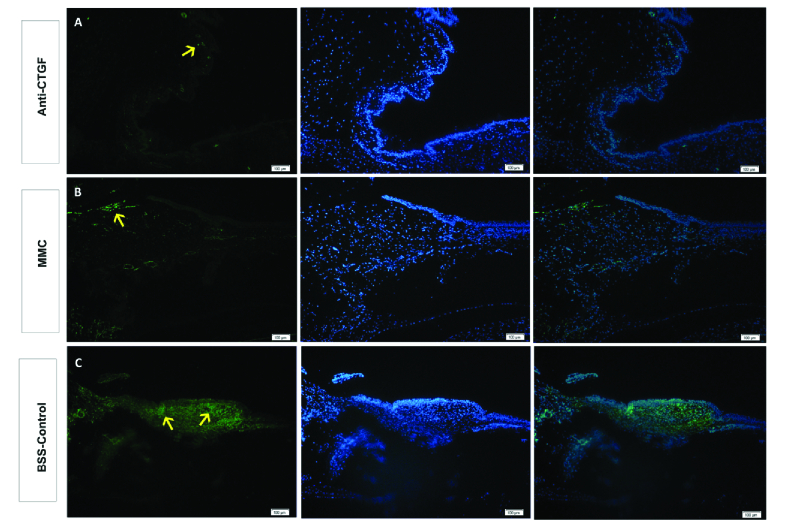
Representative photomicrographs of immune-stained trabeculectomy blebs for alpha-smooth muscle actin (α-SMA). Note significant immune reactivity for α-SMA in the control bleb (C) as compared to that in the anti-CTGF (A) and MMC (B) blebs. Yellow arrows indicate some concentrations of immune-stained cells.

**Figure 4:**
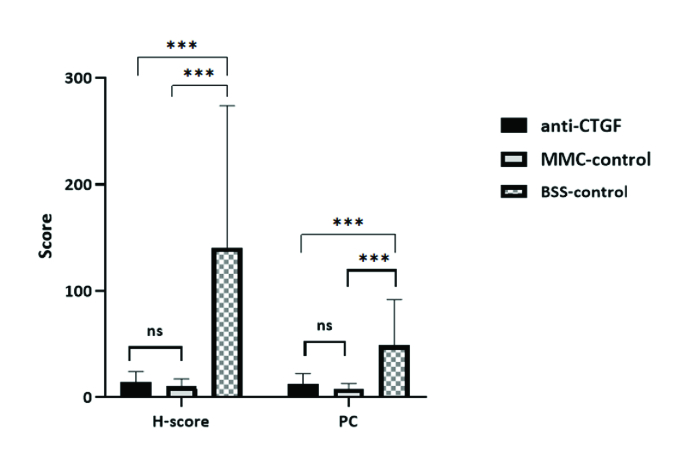
The black bar represents the mean of H-score in each group. (H-SCORE = I × PC; I, the grade of staining; and PC, the percentage of immune stained cells). PC was calculated as the ratio of green, fluorescent cells with DAPI-stained nuclei to all DAPI-stained nuclei. Significance at < 0.05, < 0.01, and < 0.001 are indicated in figures by *, **, and ***, respectively.

**Figure 5:**
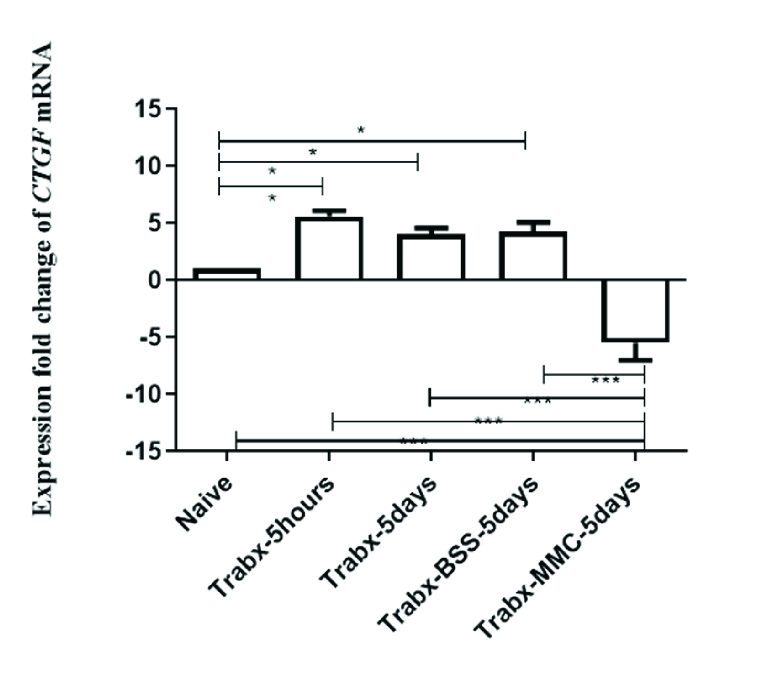
Expression fold change of CTGF mRNA. The effect of each treatment is reported as fold change with respect to control eyes. Significance at < 0.05, < 0.01, and < 0.001 are indicated in figures by *, **, and ***, respectively.

##  RESULTS

### Clinical Results

Trabeculectomy blebs persisted in all cases over the 14-day study period [Figure 1]. The mean area of the trabeculectomy blebs in the anti-CTGF, MMC, and control groups was 3.8 ± 1.45, 5.9 ± 1.4, and 3.5 ± 1.9 mm^2^, respectively. The mean bleb area was significantly larger in the MMC group as compared to the anti-CTGF and control groups (*P* = 0.025 and *P* = 0.035, respectively). The bleb area was comparable in the anti-CTGF and control eyes (*P* = 0.81) [Figure 1]. There were no intraoperative complications.

Ocular examinations in terms of corneal clarity, depth of anterior chamber, and roundness of pupils were unremarkable.

The mean IOP immediately after anesthesia was 18.33 ± 1.52, 18.66 ± 0.57, and 17.33 ± 0.57 in anti-CTG, MMC, and control groups (*P* = 0.22) respectively. IOP was significantly decreased after glaucoma filtration surgery in each study group (*P*s < 0.01). On postoperative day 14, the mean IOP was 11.0 ± 3.6, 8.6 ± 4.1, and 13.3 ± 1.5 in anti-CTGF, MMC, and BSS groups, respectively. There was no statistically significant difference among the three study groups (*P* = 0.35).

### Histopathological Results

The blue-stained collagenous tissue in the bleb occupied 13.7%, 13.5%, and 18.5% of the total bleb area in the anti-CTGF, MMC, and control groups, respectively. While the ratio of blue-stained collagenous tissue in the bleb to the total bleb area was not significantly different between the anti-CTGF and MMC groups (*P* = 0.65), it was significantly larger in the control group (*P* = 0.04) [Figure 2].

Immunohistochemical photomicrographs of α-SMA-immune reactive cells in the trabeculectomy blebs are illustrated in Figure 3. The mean PC was 12.4%, 8.1%, and 49% in the anti-CTGF, MMC, and control groups, respectively (*P* = 0.02). The mean H-SCORE of the α-SMA-immune reactive cells in the blebs was 14.6, 10.22, and 140.58 in the anti-CTGF, MMC, and control groups, respectively. Although it was not significantly different between the anti-CTGF and MMC groups (*P* = 0.87), the score was significantly higher in the control group as compared to the two other groups (*P* < 0.001) [Figure 4].

### CTGF mRNA Expression Results

The effect of trabeculectomy on CTGF mRNA expression levels was studied by quantitative real-time PCR 5 hr and five days after surgery. As shown in Figure 5, CTGF mRNA expression was increased 5 hr after trabeculectomy by more than five times of the control eye. Then, it slowly decreased and reached about four-folds of its baseline value in five days in both rabbit eyes with trabeculectomy without any injection and rabbit eyes after trabeculectomy with BSS injection. While MMC significantly reduced the CTGF mRNA expression, BSS injection did not affect it.

##  DISCUSSION

Our results showed that the antifibrotic effect of the anti-CTGF (Peprotech, NJ, USA) subconjunctival injection was comparable to MMC and significantly more than BSS in trabeculectomy blebs. To the best of our knowledge, this is the first experimental study investigating the histologic effects of an anti-CTGF antibody following glaucoma filtering surgery (GFS) in rabbits. While bleb function and morphology are important parameters in the assessment of novel medications,^[20]^ histopathology provides invaluable information on their safety and efficacy at the tissue level. In our study, the anti-CTGF antibody injection reduced the number of myofibroblasts which was comparable to the effect of MMC.

The success of trabeculectomy is limited by postoperative fibrosis at the surgical site.^[21,22]^ Fibroblasts play a critical role in the proliferation and remodeling phase of wound healing. Activated fibroblasts differentiate into myofibroblasts which is mediated by various factors including TGF-β^[23]^ and CTGF. In the rabbit model of GFS, Esson et al showed that both CTGF and TGF-β were overexpressed in the bleb area with the highest expression observed at day five post-trabeculectomy. They also showed that the injection of exogenous CTGF into the rabbit trabeculectomy bleb caused bleb scarring and failure.^[8]^ Their findings were confirmed by Yuan et al.^[12]^

The results of our experiment are in line with the study conducted by Wang et al.^[13]^ In their rabbit model of GFS, subconjunctival injection of anti-CTGF resulted in a larger bleb area and lower IOP; however, they did not investigate microstructural features of the blebs and had no histological proof on the inhibition or reduction of fibrosis in anti-CTGF-treated trabeculectomy blebs. Other human tissue studies demonstrated that inhibition of CTGF has the potential to reduce or even prevent the fibrosis process.^[24]^ Interestingly, increasing evidence suggests a role for TGF-β and CTGF in aqueous outflow resistance at the TM.^[14,16,17]^ It has been shown that CTGF is expressed in high amounts in the TM of the human eye,^[25,26]^ and may cause modifications in the TM actin cytoskeleton which leads to high IOP.^[27]^

Our results demonstrated that trabeculectomy increased CTGF mRNA expression. CTGF expression is thought to be upregulated by TGF-β and also due to the direct effect of mechanical stress beyond the TGF-β cascade.^[28]^ This increase started in the early hours after the surgery and remained high for several days. In line with our results, several other studies showed CTGF expression starting early after injury and remaining high for 24–48 hr^[29,30]^ and peaking on day five.^[8,31]^

Moreover, we noticed that MMC significantly inhibited CTGF mRNA expression on postoperative day five. Although exogenous CTGF could lead to failure of the MMC-treated bleb in the study by Esson et al,8 our result revealed robust inhibition of CTGF expression by MMC. The underlying mechanism possibly relates to MMC inhibitory effects on fibroblast proliferation. However, there is some evidence suggesting that growth-arrested fibroblasts could still release growth factors resulting in continued fibrosis by stimulating adjacent cells.^[31,32]^ The results of our experiment suggest that a single exposure to MMC significantly reduces the amount of CTGF mRNA expression even five days after surgery. Therefore, other growth factors beyond CTGF may be responsible for the continued fibrosis and scar formation after MMC injection.

Understanding the molecular biology of various ocular pathologies has led to an increasing use of monoclonal antibodies in the treatment of angiogenic and inflammatory ocular diseases.^[4]^ TGF-β is a strong stimulator of post-trabeculectomy scar and fibrosis formation through activation of fibroblasts. While experimental and early human studies on anti-TGF-β antibody on trabeculectomies proved to be effective and safe, surprisingly, the initial success was not replicated in a phase III clinical trial where the success rate of its use was only 60% as compared to 68% for placebo in preventing scarring after first-time trabeculectomy.^[5]^ The authors postulated that the lack of success in their study suggests that although TGF-β plays a key role in the wound healing process, specific targeting of TGF-β is too narrow an approach. The healing response is not sequential and is regulated by several cytokines and feedback loops. The complexity of this process renders it resistant to blockage of a single component. Therefore, it is important to investigate different monoclonal antibodies and the effect of their sequential combination on the outcomes of trabeculectomy.^[33]^ CTGF is a key downstream mediator of TGF-β-induced fibrosis and is upregulated during inflammation and wound healing. CTGF is involved in cell proliferation and migration, angiogenesis, and ECM production. The inhibition of CTGF in the liver, cardiovascular system, and respiratory system has the potential to reverse tissue remodeling and the process of fibrosis.^[9,16,21,34]^ In an experimental study, Daftarian et al demonstrated that intravitreal injection of an anti-CTGF antibody reduced fibrosis associated with choroidal neovascular membrane in comparison to intravitreal injection of bevacizumab and the control group.^[35]^

Yamanaka et al investigated the effect of CTGF inhibition on cultured subconjunctival fibroblasts (SCF). Their investigation confirmed that inhibition of CTGF resulted in reduced ECM production and fibroblast differentiation and migration. In this *in vitro* study, the proliferation of cultured SCFs was inhibited after 13 days of culture.^[36]^ In addition, myofibroblasts which are a key player in the proliferative phase of wound healing and ECM formation progressively disappear by the late stage of wound healing.^[37]^ It is not known whether these cells transform into quiescent fibroblasts without expression of α-SMA or are reduced by apoptosis.^[38]^ However, it is believed that IHC staining of these cells by α-SMA antibody in later stages cannot precisely show the difference between anti-CTGF, MMC, and control groups. Our tissue samples were obtained two weeks after the surgery to create a more detailed analysis at the tissue level. Similarly, a 14-day duration was considered the optimum time for tissue sampling in previous studies.^[29,35,39]^ However, this early sacrifice may not provide enough evidence to draw precise conclusions about the morphological and functional characteristics of the bleb.

The humanized antibody that we used in this study had 90% cross-reaction with rabbit CTGF, which could have affected its efficacy. Another limitation of this study is the lack of repeat injections in the MMC and control groups. While intraoperative MMC has been proven to have a long-term effect for at least 30 days,^[40]^ pharmacodynamics of subconjunctival anti-CTGF administration is unknown. We decided to boost the effect of the anti-CTGF by the second injection at its highest concentration on postoperative day five.^[8]^

In summary, the findings of this experimental study showed that subconjunctival injection of an anti-CTGF antibody after trabeculectomy resulted in a larger bleb and lower IOP. Moreover, histopathologic assessment demonstrated that the administration of an anti-CTGF reduced the fibrotic reaction and the number of myofibroblasts in the bleb following GFS.

##  Financial Support and Sponsorship

None.

##  Conflicts of Interest 

The authors declare no conflict of interest.

## References

[B1] Bloom P, Au L (2018). Minimally invasive glaucoma surgery (MIGS) is a poor substitute for trabeculectomy—The great debate. Ophthalmol Ther.

[B2] Bindlish R, Condon GP, Schlosser JD, D’Antonio J, Lauer KB, Lehrer R (2002). Efficacy and safety of mitomycin-C in primary trabeculectomy: Five-year follow-up. Ophthalmology.

[B3] Lockwood A, Brocchini S, Khaw PT (2013). New developments in the pharmacological modulation of wound healing after glaucoma filtration surgery. Curr Opin Pharmacol.

[B4] Rodriguez‐Una I, Azuara‐Blanco A, King AJ (2017). Survey of glaucoma surgical preferences and post‐operative care in the United Kingdom. Clin Exp Ophthalmol.

[B5] Group C-TS (2007). A phase III study of subconjunctival human anti–transforming growth factor β2 monoclonal antibody (CAT-152) to prevent scarring after first-time trabeculectomy. Ophthalmology.

[B6] Abreu JG, Ketpura NI, Reversade B, De Robertis E (2002). Connective-tissue growth factor (CTGF) modulates cell signalling by BMP and TGF-β. Nat Cell Biol.

[B7] Adler SG, Schwartz S, Williams ME, Arauz-Pacheco C, Bolton WK, Lee T, et al (2010). Phase 1 study of anti-CTGF monoclonal antibody in patients with diabetes and microalbuminuria. Clin J Am Soc Nephrol.

[B8] Esson DW, Neelakantan A, Iyer SA, Blalock TD, Balasubramanian L, Grotendorst GR, et al (2004). Expression of connective tissue growth factor after glaucoma filtration surgery in a rabbit model. Invest Ophthalmol Vis Sci.

[B9] Fan W-H, Pech M, Karnovsky MJ (2000). Connective tissue growth factor (CTGF) stimulates vascular smooth muscle cell growth and migration in vitro. Eur J Cell Biol.

[B10] Lu H, Kojima K, Battula VL, Spong S, Canizales M, Lock RB, et al (2014). Targeting connective tissue growth factor (CTGF) in acute lymphoblastic leukemia preclinical models: Anti-CTGF monoclonal antibody attenuates leukemia growth. Ann Hematol.

[B11] Wang Q, Usinger W, Nichols B, Gray J, Xu L, Seeley TW, et al (2011). Cooperative interaction of CTGF and TGF-β in animal models of fibrotic disease. Fibrogenesis Tissue Repair.

[B12] Yuan HP, Li XH, Yang BB, Shao ZB, Yan LP (2009). [Expression of connective tissue growth factor after trabeculectomy in rabbits]. Zhonghua Yan Ke Za Zhi.

[B13] Wang JM, Hui N, Fan YZ, Xiong L, Sun NX (2011). Filtering bleb area and intraocular pressure following subconjunctival injection of CTGF antibody after glaucoma filtration surgery in rabbits. Int J Ophthalmol.

[B14] Browne JG, Ho SL, Kane R, Oliver N, Clark AF, O'Brien CJ, et al (2011). Connective tissue growth factor is increased in pseudoexfoliation glaucoma. Invest Ophthalmol Vis Sci.

[B15] Fleenor  DL, Shepard  A, Jacobson  N, Pang I-H, Clark AF

[B16] Shepard AR, Pang I-H

[B17] Taylor AW (2012). Primary open-angle glaucoma: A transforming growth factor-β pathway–mediated disease. Am J Pathol.

[B18] Motevasseli T, Daftarian N, Kanavi MR, Ahmadieh H, Bagheri A, Hosseini SB, et al (2017). Ocular safety of intravitreal connective tissue growth factor neutralizing antibody. Curr Eye Res.

[B19] Fedchenko N, Reifenrath J (2014). Different approaches for interpretation and reporting of immunohistochemistry analysis results in the bone tissue–a review. Diagnostic Pathol.

[B20] Esfandiari H, Pakravan M, Loewen NA, Yaseri M (2017). Predictive value of early postoperative IOP and bleb morphology in Mitomycin-C augmented trabeculectomy. F1000Research.

[B21] Mead AL, Wong TT, Cordeiro MF, Anderson IK, Khaw PT (2003). Evaluation of anti-TGF-β2 antibody as a new postoperative anti-scarring agent in glaucoma surgery. Invest Ophthalmol Vis Sci.

[B22] Seibold LK, Sherwood MB, Kahook MY (2012). Wound modulation after filtration surgery. Surv Ophthalmol.

[B23] Wipff P-J, Rifkin DB, Meister J-J, Hinz B (2007). Myofibroblast contraction activates latent TGF-β1 from the extracellular matrix. J Cell Biol.

[B24] Lipson KE, Wong C, Teng Y, Spong S (2012). CTGF is a central mediator of tissue remodeling and fibrosis and its inhibition can reverse the process of fibrosis. Fibrogenesis Tissue Repair.

[B25] Iyer P, Maddala R, Pattabiraman PP, Rao PV (2012). Connective tissue growth factor–mediated upregulation of neuromedin U expression in trabecular meshwork cells and its role in homeostasis of aqueous humor outflow. Invest Ophthalmol Vis Sci.

[B26] Kuespert S, Junglas B, Braunger BM, Tamm ER, Fuchshofer R (2015). The regulation of connective tissue growth factor expression influences the viability of human trabecular meshwork cells. J Cell Mol Med.

[B27] Junglas B, Kuespert S, Seleem AA, Struller T, Ullmann S, Bösl M, et al (2012). Connective tissue growth factor causes glaucoma by modifying the actin cytoskeleton of the trabecular meshwork. Am J Pathol.

[B28] Schild C, Trueb B (2002). Mechanical stress is required for high-level expression of connective tissue growth factor. Exp Cell Res.

[B29] Daftarian N, Bayeghi O, Rezaei Kanavi M, Ahmadieh H (2019). Effects of intravitreal connective tissue growth factor neutralizing antibody on the epiretinal membrane formation; an experimental study. Invest Ophthalmol Vis Sci.

[B30] Igarashi A, Okochi H, Bradham D, Grotendorst GR (1993). Regulation of connective tissue growth factor gene expression in human skin fibroblasts and during wound repair. Mol Cell Biol.

[B31] Daniels JT, Occleston NL, Crowston JG, Khaw PT (1999). Effects of antimetabolite induced cellular growth arrest on fibroblast-fibroblast interactions. Exp Eye Res.

[B32] Occleston NL, Daniels JT, Tarnuzzer RW, Sethi KK, Alexander RA, Bhattacharya SS, et al (1997). Single exposures to antiproliferatives: Long-term effects on ocular fibroblast wound-healing behavior. Invest Ophthalmol Vis Sci.

[B33] Sherwood MB (2006). A sequential, multiple-treatment, targeted approach to reduce wound healing and failure of glaucoma filtration surgery in a rabbit model (an American Ophthalmological Society thesis). Trans Am Ophthalmol Soc.

[B34] Yu-Wai-Man C, Khaw PT (2015). Developing novel anti-fibrotic therapeutics to modulate post-surgical wound healing in glaucoma: big potential for small molecules. Expert Rev Ophthalmol.

[B35] Daftarian N, Rohani S, Kanavi MR, Suri F, Mirrahimi M, Hafezi-Moghadam A, et al (2019). Effects of intravitreal connective tissue growth factor neutralizing antibody on choroidal neovascular membrane-associated subretinal fibrosis. Exp Eye Res.

[B36] Yamanaka O, Saika S, Ikeda K, Miyazaki K-i, Kitano A, Ohnishi Y (2008). Connective tissue growth factor modulates extracellular matrix production in human subconjunctival fibroblasts and their proliferation and migration in vitro. Jpn J Ophthalmol.

[B37] Li B, Wang JH-C (2011). Fibroblasts and myofibroblasts in wound healing: Force generation and measurement. J Tissue Viability.

[B38] Darby IA, Laverdet B, Bonté F, Desmoulière A (2014). Fibroblasts and myofibroblasts in wound healing. Clin Cosmet Investig Dermatol.

[B39] Kuiper EJ, Roestenberg P, Ehlken C, Lambert V, van Treslong-de Groot HB, Lyons KM, et al (2007). Angiogenesis is not impaired in connective tissue growth factor (CTGF) knock-out mice. J Histochem Cytochem.

[B40] Khaw PT, Sherwood MB, MacKay SL, Rossi MJ, Schultz G (1992). Five-minute treatments with fluorouracil, floxuridine, and mitomycin have long-term effects on human Tenon's capsule fibroblasts. Arch Ophthalmol.

